# Bio-Refinery of Organics into Value-Added Biopolymers: Exploring the Effects of Hydraulic Retention Time and Organic Loading Rate on Biopolymer Harvesting from a Biofilm-Based Process

**DOI:** 10.3390/toxics13030183

**Published:** 2025-02-28

**Authors:** Qingna Shang, Lin Li, Yi Zhang, Xueqing Shi, Harsha Ratnaweera, Dong-Hoon Kim, Haifeng Zhang

**Affiliations:** 1 National and Local & Joint Engineering Research Center for Urban Sewage Treatment and Resource Recycling, School of Environmental and Municipal Engineering, Qingdao University of Technology, 11 Fushun Road, Qingdao 266033, China; xiayu7200@163.com (Q.S.); li_lin08@163.com (L.L.); royce_zy2000@163.com (Y.Z.); shixq85@163.com (X.S.); harsha.ratnaweera@nmbu.no (H.R.); 2 Department of Smart City Engineering, Inha University, 100 Inharo, Michuhol-gu, Incheon 22212, Republic of Korea; dhkim77@inha.ac.kr

**Keywords:** alginate like exopolymers (ALEs), extracellular polymeric substances (EPSs), microbial community, moving bed biofilm reactor (MBBR), wastewater reclamation

## Abstract

This study aimed to examine the impacts of hydraulic retention time (HRT) and organic loading rate (OLR) on the alginate-like exopolymers’ (ALEs) recovery potential from a biofilm-based process. A lab-scale moving bed biofilm reactor (MBBR) was operated under different HRT (12.0, 6.0, and 2.0 h) and OLR (1.0, 2.0, and 6.0 kg COD/m^3^/d) conditions. The results demonstrated that the reduction in HRT and increase in OLR had remarkable effects on enhancing ALE production and improving its properties, which resulted in the ALE yield increasing from 177.8 to 221.5 mg/g VSS, with the protein content rising from 399.3 to 494.3 mg/g ALE and the enhanced alginate purity by 39.8%, corresponding to the TOC concentration increasing from 108.3 to 157.0 mg/g ALE. Meanwhile, to illustrate different ALE recovery potentials, microbial community compositions of the MBBR at various operational conditions were also assessed. The results showed that a higher relative abundance of EPS producers (29.86%) was observed in the MBBR with an HRT of 2.0 h than that of 12.0 h and 6.0 h, revealing its higher ALE recovery potential. This study yields crucial results in terms of resource recovery for wastewater reclamation by providing an effective approach to directionally cultivating ALEs.

## 1. Introduction

Recovering biomaterials, such as alginate-like exopolymers (ALEs), sulfated polysaccharides (SPs), tryptophan, and so on, from excess biomass generated in wastewater treatment processes has attracted great interest for research and applications [1,2]. Among them, ALEs, major constituents of extracellular polymeric substances (EPSs), recovered from microbial biomass have been considered as high value-added biomaterials with wide application potentials [3]. For example, ALE was evaluated as a coating material and a bio-sorbent for the removal of refractory pollutants [4]. Moreover, several researches have also reported that ALE shows promise for application in the food, paper, construction, immobilization of microorganisms, and agriculture industries for its biodegradability, non-toxicity, and similarity with commercial chemical polymers [1,3,5,6]. For this reason, market conditions are especially favorable for the production of ALEs from excess biomass. For example, a total of 85 kilotons of ALEs are expected annually to be retrieved from ten individual wastewater treatment plants (WWTPs) in the Netherlands, and they are estimated to produce 170 million euros by 2030 [7]. Under this circumstance, enhancing the ALE recovery from excess biomass and improving its properties is significant for promoting a paradigm shift of WWTPs from a waste stream to a resource recovery factory.

Several researches have shown that the ALE formation was significantly affected by operating conditions of wastewater treatment processes, including temperature, influent carbon source, sludge retention time (SRT), etc. [8,9,10]. For example, Li et al. (2022) [10] found that the yields of ALEs could be enhanced from approximately 130.0 mg/g VSS to 303.3 mg/g VSS with the decrease in temperature from 24 °C to 12 °C. Moreover, Ferreira dos Santos et al. (2022) [9] demonstrated that using acetate as the influent substrate was favorable to enrich ALE formation in the aerobic granular sludge (AGS), where the ALE yield extracted from the AGS system with acetate as a carbon source was 2.3 times that extracted from the AGS system in the presence of propionate as substrate. Although the ALE production could be enhanced by adjusting the above-mentioned operational conditions, the implementation of relevant strategies is frequently not cost-effective and not feasible in practice [11]. Therefore, effective and sustainable approaches need to be developed and evaluated for the recovery of highly valuable biopolymers from wastewater treatment processes. Moreover, it is reported that the granulation rate of AGS, as well as the composition of microbial communities within the granules, may be considerably influenced by the organic loading rate (OLR) [12,13]. For example, Yang et al. (2014) [12] revealed that numerous strains such as *Pseudomonas*, *Clostriduim*, *Thauera*, and *Arthrobacter* could be enriched in the formed granules when the OLR increased from 4.4 to 17.4 kg COD/m^3^/d, which functioned as the secretion of extracellular cyclic diguanylate (c-di-GMP) and EPS production. Therefore, it is expected that the ALE formation might be influenced by feed OLR. However, the investigation of the influence of OLR on ALE recovery from municipal wastewater is still limited.

Regarding municipal wastewater, chemical oxygen demand (COD) levels in China’s municipal wastewater typically range from 200 to 400 mg/L, corresponding to an average low OLR in wastewater [14]. Optimizing the operational condition of the wastewater treatment process, such as applying a low hydraulic retention time (HRT), could improve the OLR and provide an advantage in the screening of EPS-forming microbes. In addition, flocculent sludge (e.g., conventional activated sludge) and biofilm-based biological processes (e.g., the moving bed biofilm reactor (MBBR) and granular sludge) have been widely applied in municipal wastewater and industrial wastewater treatment [15,16,17]. Among them, the MBBR process has been identified as a popular biological treatment process due to its high microbial activity, excellent environmental adaptability, and great capacity to endure high organic loading [18]. Therefore, it is expected to improve the biopolymer recovery potential from excess biomass of the MBBR process through optimizing HRT and OLR conditions. However, the optimal operational condition, including the recovery potential, purity of ALEs, and the underlying mechanism of this optimal strategy, is still limited.

For the reason described above, this study was initiated to examine the impact of HRT and OLR on the ALE recovery potential from municipal wastewater through a biofilm-based process. The treatment performance, ALE recovery potential, and properties analyses were assessed. Moreover, an additional assessment of the microbial community composition was further conducted to reveal the underlying mechanism of ALE formation.

## 2. Materials and Methods

### 2.1. System Setup

A cylindrical MBBR with an effective volume of 2.0 L was used in this study. The system was configured to facilitate aerobic conditions, and the process of fluidization of the bio-carriers was ensured by incorporating a microporous aeration disk and a mechanical stirrer with a speed of 200 rpm into the system. The aeration was supplied with a flow rate of 1 L/min by the air diffusers located at the bottom of the reactor. The influent was introduced through a peristaltic pump from the bottom of the reactor, while the effluent was discharged through the overflow weir positioned at the top of the reactor. The high-density polyethylene K1 carriers were used in this study, whose volumetric filling ratio (carrier volume to effective volume ratio) was 25%, specific surface area was 760 m^2^/m^3^, and diameter, as well as height, were both 1 cm. During the system start-up phase, the seeding sludge (VSS/TSS = 0.7) taken from an aerobic tank in a municipal WWTP in Qingdao, China, was utilized with an inoculation concentration of 100 mg TSS/L.

### 2.2. Wastewater Characteristics and Operational Conditions

As illustrated in Table 1, the composition of synthetic municipal wastewater and the operational conditions of the MBBR have been thoroughly delineated. The concentrations of COD, NH_4_^+^-N, and phosphorus were set to 500.0, 25.0, and 5.0 mg/L, respectively. The CH_3_COONa, NH_4_Cl, and KH_2_PO_4_ were used as the carbon source, nitrogen source, and phosphorus source, respectively. A trace element solution was also used according to Zhang et al. (2022) [18]. During the entire experiment, the MBBR was consistently maintained at various HRTs of 12.0 h, 6.0 h, and 2.0 h, respectively, which corresponded to organic loading rates (OLR) of 1.0 kg Chemical Oxygen Demand (COD)/m^3^/d, 2.0 kg COD/m^3^/d, and 6.0 kg COD/m^3^/d, respectively. The concentration of dissolved oxygen (DO) was maintained within the range of 5.0–6.0 mg/L. The temperature was controlled between 22.0 and 25.0 °C, and the pH was kept within the 7.0–8.0 range.

### 2.3. Analytical Methods

General parameters, such as COD, NH_4_^+^-N, NO_3_^−^-N, NO_2_^−^-N, total nitrogen (TN), mixed liquid suspended solids (MLSS), mixed liquid volatile suspended solids (MLVSS), and sludge volume index (SVI_30_), were measured in accordance with the APHA standard methods [19]. The ALE was extracted by the process of modified high temperature-sodium carbonate, and the detailed method was described in Zhang et al. (2024) [1]. The concentrations of proteins (PN) and polysaccharides (PS) were analyzed through the utilization of the modified Lowry–Folin method [20] and the phenol–sulfuric acid method [21], respectively. An alginate equivalent analysis was performed to evaluate the purity of the ALE sample through the phenol–sulfuric acid method, and the commercial alginate was utilized as standard [22]. In order to further investigate the organic proportions of the ALE, the total organic carbon (TOC) was determined by analyzing it using a TOC analyzer (Analytica Jena AG, Jena, Germany). The characterization of the surface chemical functional groups of the ALE samples was accomplished by means of Fourier-transformation infrared spectroscopy (FT-IR, Perkin Elmer, Waltham, MA., USA). First, 2 mg dried samples and 98 mg KBr were measured at the wave number range of 4000–400 cm^−1^. UV-visible absorbance measurements (DR5000, HACH, Loveland, CO, USA) were also carried out to detect the humification and aromaticity of the ALE from 800 nm to 200 nm. All measurements were carried out in triplicate, and the experimental results were presented as mean ± standard deviation.

At steady state, the collection of biofilm and suspended biomass samples was undertaken for the purposes of DNA extraction, with these samples then being subjected to a mixed liquid process within the MBBR. The Power Soil DNA extraction kit (OMEAG-soil, Omega Bio-Tek, Norcross, GA, USA) was utilized for the extraction of DNA from the samples. The V3-V4 region of the bacterial 16S rRNA gene was amplified by PCR using primers 338F (5′-ACTCCTACGGGAGGCAGCAG-3′) and 806R (5′-GGACTACHVGGGTWTCTAAT-3′). The amplicon sequencing was conducted on an Illumina MiSeq platform (Majorbio, Shanghai, China) and then quality-filtered and merged by fastp (v 0.19.6) and FLASH (v 1.2.7), respectively.

## 3. Results and Discussion

### 3.1. MBBR Performance

#### 3.1.1. Treatment Performance

**Figure 1:**
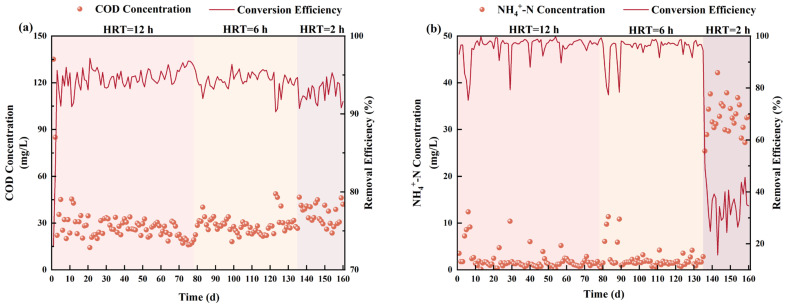
MBBR performances regarding COD and nitrogen species removal under different HRT conditions: (**a**) COD and (**b**) NH_4_^+^-N.

The treatment performance of the MBBR system under different OLR and HRT conditions is illustrated in Figure 1 and Table 2. In general, as shown in Figure 1a, it is found that a stable and excellent organic removal rate could be observed in the MBBR system during the whole operational period, with the average effluent concentration and removal rate of 28.99 ± 4.22 mg/L and 94.5 ± 0.9%, respectively. It is indicated that the variation of HRT and OLR had little effect on organics removal in the MBBR. As depicted in Figure 1b, the removal efficiency of NH_4_^+^-N remained at a high level when HRT = 12 h and HRT = 6 h, with the removal efficiency of 97.2 ± 1.4% and 96.8 ± 1.2%, respectively. Then, a reduction in HRT from 6.0 h to 2.0 h was observed to negatively impact the removal efficiency of NH_4_^+^-N. Consequently, the effluent NH_4_^+^-N concentration increased to 32.04 ± 3.08 mg/L, with a corresponding removal efficiency of 36.4 ± 5.8%. This phenomenon could be attributed to the heightened sensitivity of autotrophic microorganisms to environmental fluctuations and the inherently low metabolic kinetics of the nitrifiers. A similar result was reported by Zhang et al. (2024) [5], who found that a relatively low NH_4_^+^-N removal efficiency of 12.6 ± 0.8% was obtained in a biofilm-based reactor under a low HRT of 1.0 h. In other words, the achievement of nitrification blockage might be conducive to achieve low energy removal (i.e., anammox process) [23] or high-value recovery of ammonia nitrogen (bio-refinery for an amino acid) [24].

#### 3.1.2. Effluent Biomass Characteristics and Settleability Analyses

**Figure 2:**
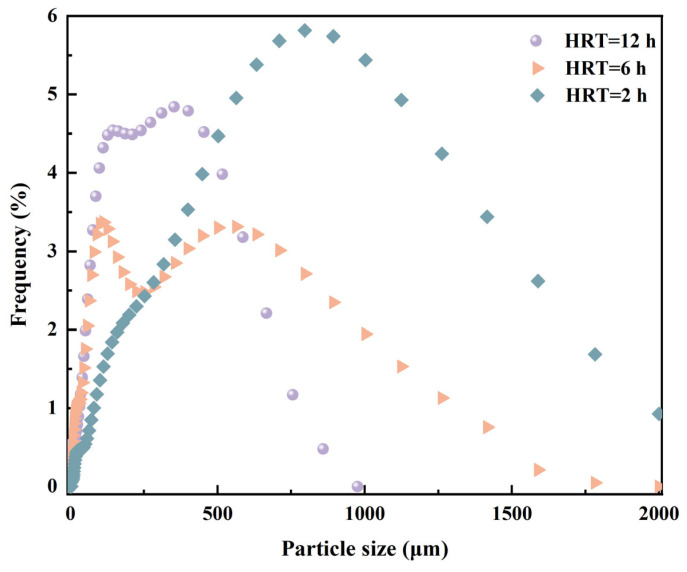
Particle size distribution of suspended biomass of the MBBR under different HRT conditions.

Interestingly, the SVI_30_ of the suspended biomass at the HRT of 2.0 h (86.9 mL/g) was significantly lower than that of HRT = 6.0 h (165.9 mL/g) and HRT = 12.0 (no obvious settling after 30 min) (Table 2). It is indicated that the settleability of suspended biomass was improved with the application of the high-rate MBBR, which might be conducive to biomass recovery for biopolymer harvesting. This phenomenon might be explained by the fact that self-flocculation of a suspended biomass might occur in the MBBR under a low HRT condition. Moreover, the particle size distribution of the suspended biomass obtained from the MBBR at different HRT conditions was assessed to further reveal the sludge characteristics. As illustrated in Figure 2, the mean particle sizes of the suspended biomass exhibited an increasing trend with the reduction of HRT, and the mean particle sizes under the HRT conditions of 12 h, 6 h, and 2 h were 352.1 μm, 563.7 μm, and 796.2 μm, respectively. This finding was in accordance with the results of SVI_30_, which further demonstrated that self-flocculation was effectively achieved to form large particles when the HRT was shortened.

### 3.2. Biopolymer Production and Property Analyses

#### 3.2.1. Biopolymer Production

**Figure 3:**
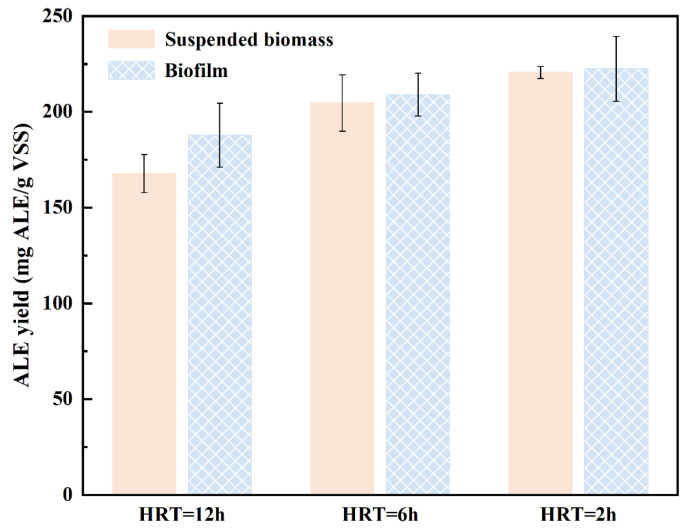
The ALE yields of the MBBR at different HRT conditions.

To further reveal the effects of HRT and OLR on the ALE recovery potential of the MBBR system, the biomass samples including biofilm and suspended biomass were collected for ALE extraction under a steady state. The ALE yields obtained from suspended biomass and biofilm under different HRT conditions are depicted in Figure 3. In general, the ALE yield of the MBBR system was in the range of 167.73–222.37 mg/g VSS, which was meaningfully higher than that of typical activated sludge (90–190 mg/g VSS) [25]. This might be explained by the fact that the biocenosis of biofilm is completely different from that in typical activated sludge, and the formation of biofilm is related to the strong secretion of EPSs. Moreover, an increasing trend of ALE production could be observed with the decrease in HRT from 12.0 h to 6.0 h and 2.0 h. It has been reported that under harsh environments, microbes tend to secrete an increased amount of EPSs, which increases the ALE production of the MBBR system [18]. It is indicated that the HRT and OLR have great effects on the ALE production. In addition, a decreasing trend of the difference between the ALE production of biofilm and suspended biomass could be observed with the decrease in HRT, with the differences of 20.07 mg/g ALE, 4.34 mg/g ALE, and 1.85 mg/g ALE, respectively (Figure 3). This may be attributed to an increase in OLR, the vigorous metabolism of microorganisms, and the acceleration of biofilm regeneration. A proportion of suspended biomass is derived from biofilm, thus resulting in a certain similarity and homology of microbial communities between biofilm and suspended biomass [26]. Therefore, it is indicated that an excellent ALE recovery potential could be observed from both biofilm and suspended biomass, which was obtained from the high-rate MBBR process.

#### 3.2.2. ALE Composition and Property Analyses

**Figure 4:**
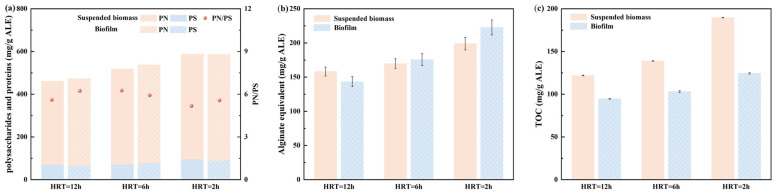
Component analyses of ALEs; (**a**) polysaccharides and proteins; (**b**) alginate equivalent; (**c**) TOC.

As illustrated in Figure 4a, protein (PN) was the predominant component of ALEs extracted from suspended biomass and biofilm with the PN ranges of 391.23–496.56 mg/g ALE under different HRT conditions, and the highest PN concentration (496.56 mg/g ALE) was obtained from the ALE recovered from the MBBR at the HRT of 2.0 h. Moreover, an increasing trend of PN contents in the ALE recovered from the MBBR at different HRT conditions could be observed with the decrease in HRT, suggesting that the variation of HRT and OLR might show great influence on the PN content of ALEs. In addition, there was no obvious difference in the polysaccharide (PS) content (69.94–95.17 mg/g ALE) of the ALE recovered from the MBBR at the different HRT conditions. In addition, the composition of the ALE was investigated by utilizing commercial alginate as the standard for quantification of the alginate equivalent in the ALE [11]. As shown in Figure 4b, the average alginate equivalents of the ALE recovered from the MBBR increased from 143.28 (HRT = 12.0 h) to 175.51 (HRT = 6.0 h) and 222.68 (HRT = 2.0 h), indicating that the decrease in HRT might be favorable for the high purification of alginate. Similarly, the TOC of the ALE increased with decreasing HRT, ranging from 122 to 189.6 mg/g ALE, and the maximum value was attained at HRT = 2 h (Figure 4c). In all, the MBBR under low HRT and high OLR conditions might be possible to achieve directional cultivation to promote the biopolymer/ALE production and improve its compositions. Therefore, the feasible applications or economic benefits for highly valuable biopolymer recovery could be improved.

**Figure 5:**
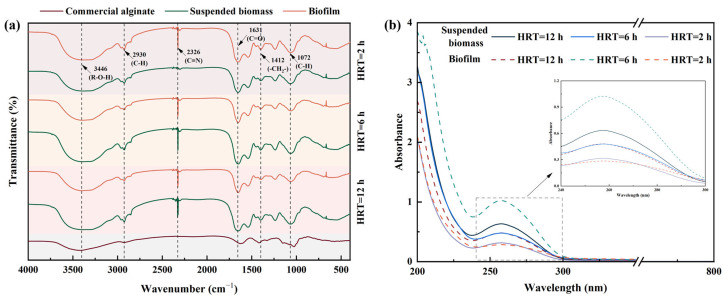
Spectral analyses of different ALEs and commercial alginate under different HRT conditions; (**a**) FT-IR spectra; (**b**) UV spectra.

The composition of functional groups is of significant importance in the analysis of the chemical properties of ALEs. It can be employed as a principal criterion for the evaluation of the potential for ALEs to supplant commercial alginate. The specific functional groups available at commercial alginate and ALEs obtained in suspended biomass and biofilm under different HRT conditions were identified by FT-IR analysis, with the results presented in Figure 5a. Various functional groups were observed on the surface of ALEs and commercial alginate, including the –CH_2_– stretching vibration point at 1412 cm^−1^, C=O at 1631 cm^−1^, and –OH at 3446 cm^−1^, which illustrated that the functional groups of ALEs obtained in suspended biomass and biofilm were similar to those of the commercial alginate. 

The presence of a broad and rounded absorption band centered around 3446 cm^−1^ was indicative of extracted ALEs, which was primarily attributable to the stretch vibrations of hydroxyl groups in carboxylic acids and sugars [27]. The C=O stretching vibration pointed at 1631 cm^−1^ suggested that the PN compositions in ALEs obtained in suspended biomass had a difference from that in biofilm. It indicated that the formation of ALEs in suspended biomass involved a greater proportion of protein-like substances [28]. The alterations in the biofilm spectra were observed with decreased HRT, which may reflect the enhanced synthesis of extracellular polymer (EPS) and intensified microbial metabolic processes within the biofilm [1]. In the biofilm with HRT = 2 h, the presence of more distinctive peaks associated with polysaccharides and proteins may be discerned. These include C–H and –CH_2_– bending vibration peaks at 1412 cm⁻^1^, 2930 cm^−1^, and 1072 cm⁻^1^, which indicates the potential for a greater accumulation of polysaccharides and proteins to occur in the biofilm with a shortened HRT [29]. It indicated that the ALEs obtained from both suspended biomass and biofilm under extended HRT had the potential to serve as an alternative to commercial alginate.

In addition, ALEs obtained from suspended biomass and biofilm under HRT = 12 h, HRT = 6 h, and HRT = 2 h displayed by UV-visible spectra are presented in Figure 5b and Table 3. The presence of a shoulder was detected at a wavelength ranging from 240 to 280 nm in the ALEs derived from both suspended biomass and biofilm. This observation was commonly attributed to a π → π* electron transition, a process commonly observed in aromatic and poly-aromatic compounds [30]. It could be attributed to the presence of nucleic acids, proteins, or other UV-absorbing substances. The absorbance of the ALEs obtained from biofilm exhibited minimal variation with HRT and was observed to be lower than that of the ALEs obtained from the suspended biomass. As illustrated in Table 3, the lowest values of E_2_/E_3_ and E_2_/E_4_ of ALEs obtained from suspended biomass were observed when HRT = 6 h, while that of ALEs derived from biofilm at HRT = 2 h exhibited the lowest values of E_4_/E_6_. It demonstrated that the ALEs exhibited elevated molecular weight, an enhanced organic molecular condensation degree, and augmented aromatic structure content under low HRT conditions, indicating a more intricate structural configuration [31].

### 3.3. Microbial Community Diversity and Composition Analyses

#### 3.3.1. Microbial Community Diversity

As illustrated in Table 4, the coverage index of the MBBR under different HRT conditions exceeded 0.99, suggesting that the depth of DNA sequencing was sufficient to cover most of the microbial populations in the samples. Moreover, an increasing trend of the Simpson index of the MBBR under different HRT conditions was observed, indicating that the species richness of the MBBR declined with the decrease in HRT. Meanwhile, community diversities of the MBBR also decreased with the drop in Shannon and Chao1 as well as the ACE index. Thus, the changes in HRT had great effects on the microbial community of the MBBR. The decrease in community richness and diversity of the MBBR might be attributed to the washing out of the microbes with slow growth rates and poor adaptability to the environment under a low HRT condition.

#### 3.3.2. Microbial Community Composition

**Figure 6:**
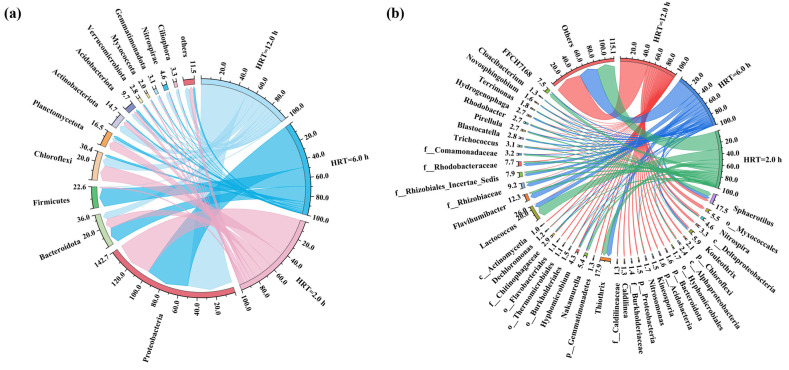
The microbial community composition of the MBBR under different HRT conditions: (**a**) phylum and (**b**) genus level.

The relative abundance of the taxon assignments at the phylum and genus levels for the bacterial community composition of the MBBR under different HRT conditions is illustrated in Figure 6. On the phylum level, 9, 10, and 7 major phyla (relative abundance > 1.0%) were observed in the MBBR for the HRT of 12.0 h, 6.0 h, and 2.0 h, respectively. Proteobacteria, Bacteroidota, Chloroflexi, Planctomycetota, and Acidobacteriota were the dominant phyla in the MBBR under different HRT conditions, which was consistent with previous studies [32,33,34]. Proteobacteria was the most abundant phylum in all samples, presenting a descending trend from the initial (51.29%) to 48.58% and 42.80% with the decrease in HRT from 12.0 h to 2.0 h. The relative abundance of Bacteroidota remained higher in the MBBR with the HRT of 2.0 h (17.11%) than that of 12.0 h (7.84%). These phyla are among the most abundant heterotrophic bacterial groups and play a key role in the degradation of organic matter, which may lead to an excellent COD removal rate of the MBBR under different HRT conditions [35].

On the genus level, a total of 26, 17, and 13 dominant genera (relative abundance > 1.0%) were detected in the MBBR with the HRTs of 12.0 h, 6.0 h, and 2.0 h, respectively (Figure 6b). The MBBR system with the HRT of 2.0 h showed a higher relative abundance of the EPS producer as compared with HRTs of 12.0 h and 6.0 h, a finding that has the potential to enhance the ALE recovery potential of the system. For instance, the genera of *Sphaerotilus* [36], *Lactococcus* [8], *Flavihumibacter* [37], and *Rhodobacter* [38] were enriched in the MBBR system with the shortened HRT. The HRT of 2.0 h exhibited a total relative abundance of 29.86%, which was higher than that of the HRT of 6.0 h (23.82%) and 12.0 h (4.80%). Moreover, the *Trichococcus* bacteria was identified as an EPS degrader [39], which was enriched in the MBBR system at the HRT of 6.0 h with a higher relative abundance of 2.88% as compared with the HRTs of 12.0 h (0.10%) and 2.0 h (0.88%). This might be the reason for the lower ALE recovery potential of the MBBR with an HRT of 6.0 h than that of HRT = 2.0 h. Furthermore, a descending trend of the relative abundance of nitrifiers (e.g., *Nitrospira* and *Nitrosomonas*) was observed with the decrease in HRT, with a total relative abundance of 5.27% (HRT = 12.0 h), 0.62% (HRT = 6.0 h), and 0.23% (HRT = 2.0 h), respectively. This finding provides evidence that the blockage of nitrification could be successfully achieved by washing out nitrifiers under low HRT and high OLR conditions, which is conducive to realizing low energy removal of NH_4_^+^-N (i.e., anammox process) or nutrient recovery.

## 4. Conclusions

The impacts of HRT and OLR on the ALE recovery potential of the MBBR process have been explored. The ALE production was enhanced from 177.8 to 221.5 mg/g VSS with the reduction of HRT and increase in OLR. Moreover, the ALE composition analyses demonstrated that the reduction of HRT had great effects on the ALE compositions, which resulted in the average PN content of ALEs increasing from 399.3 to 494.3 mg/g ALE and the enhancement of alginate purity by 39.8%, corresponding to the TOC concentration increasing from 108.3 to 157.0 mg/g ALE. Moreover, a higher relative abundance of EPS producers (29.86%) was observed in the MBBR with HRT of 2.0 h as compared with HRT = 12.0 h and HRT = 6.0 h, which might contribute to its higher ALE production.

## Figures and Tables

**Table 1:** Wastewater characterization and operating conditions of the MBBR.

Indices	Parameter	Value
Influent wastewater	SCOD (mg/L)	500.0
	NH_4_^+^-N (mg/L)	25.0
	Phosphorus (mg/L)	5.0
	Organic loading rate (OLR) (kg COD·m^−3^·d^−1^)	1.0–6.0
Operating condition	Hydraulic retention time (HRT) (h)	2.0–12.0
	DO (mg/L)	5.0–6.0
	Temperature (°C)	22.0–25.0
	pH	7.0–8.0
	Sludge retention time (d)	Not control
	Filling ratio (%) (*v*/*v*)	25.0

**Table 2:** Summary of treatment performance and effluent quality of the MBBR.

HRT (h)	Parameter	Concentration (mg/L)	Reduction (%)
12	SCOD	25.79 ± 4.45	95.9 ± 1.0
	NH_4_^+^-N	1.44 ± 0.96	97.2 ± 1.4
	Effluent SS	395.6 ± 12.7	-
	SVI_30_ of effluent SS	-	-
6	SCOD	27.32 ± 3.69	94.8 ± 0.6
	NH_4_^+^-N	1.55 ± 0.55	96.8 ± 1.2
	Effluent SS	482.3 ± 26.4	-
	SVI_30_ of effluent SS	165.9 ± 6.7	-
2	SCOD	33.87 ± 4.54	92.9 ± 1.3
	NH_4_^+^-N	32.04 ± 3.08	36.4 ± 5.8
	Effluent SS	690.5 ± 34.7	-
	SVI_30_ of effluent SS	86.9 ± 4.3 **	-

**: *p* < 0.05.

**Table 3:** Special absorbance ratio of ALEs extracted from suspended biomass and biofilm under different HRT conditions.

HRT (h)	Source	E2/E3	E2/E4	E4/E6
12	Suspended biomass	38.80	45.10	2.33
	Biofilm	29.80	51.14	4.00
6	Suspended biomass	15.34	20.94	2.40
	Biofilm	35.00	47.25	2.50
2	Suspended biomass	29.10	33.00	1.67
	Biofilm	7.74	9.88	4.00

**Table 4:** Microbial diversity indices of the AGS with various sizes.

Diversity Index	HRT = 12.0 h	HRT = 6.0 h	HRT = 2.0 h
OTUs	1665	1373	997
ACE	1743.6	1417.0	1104.6
Chao1	1689.5	1399.3	1109.7
Shannon	5.75	5.31	4.07
Simpson	0.01	0.02	0.06
Coverage	0.99	0.99	0.99

## Data Availability

Dataset available on reasonable request.

## Abbreviations

The following abbreviations are used in this manuscript:

HRT	Hydraulic retention time
ALEs	Alginate like exopolymers
OLR	Organic loading rate
EPSs	Extracellular polymeric substances
MBBR	Moving bed biofilm reactor
AGS	Aerobic granular sludge
SP	Sulfated polysaccharide
WWTP	Wastewater treatment plant
SRT	Sludge retention time
COD	Chemical oxygen demand
SCOD	Soluble chemical oxygen demand
MLSS	Mixed liquid suspended solids
MLVSS	Mixed liquid volatile suspended solids
TOC	Total organic carbon
PN	Protein
PS	Polysaccharide
NH_4_^+^-N	Ammonia nitrogen
NO_3_^−^-N	Nitrate nitrogen
NO_2_^−^-N	Nitrite nitrogen
TN	Total nitrogen
